# Structural basis for substrate specificity and catalysis of α1,6-fucosyltransferase

**DOI:** 10.1038/s41467-020-14794-z

**Published:** 2020-02-20

**Authors:** Ana García-García, Laura Ceballos-Laita, Sonia Serna, Raik Artschwager, Niels C. Reichardt, Francisco Corzana, Ramon Hurtado-Guerrero

**Affiliations:** 1 Institute of Biocomputation and Physics of Complex Systems (BIFI), University of Zaragoza, Mariano Esquillor s/n, Campus Rio Ebro, Edificio I+D, Zaragoza, Spain; 2 CIC biomaGUNE, Paseo Miramón 182, San Sebastián, Spain; 3 Basque Research and Technology Alliance (BRTA), Paseo Miramón 182, San Sebastian, Spain; 4 CIBER-BBN, Paseo Miramón 182, San Sebastian, Spain; 5 Departamento de Química, Universidad de La Rioja, Centro de Investigación en Síntesis Química, E-26006 Logroño, Spain; 6 Copenhagen Center for Glycomics, Department of Cellular and Molecular Medicine, School of Dentistry, University of Copenhagen, Copenhagen, Denmark; 7 Laboratorio de Microscopías Avanzada (LMA), University of Zaragoza, Mariano Esquillor s/n, Campus Rio Ebro, Edificio I+D, Zaragoza, Spain; 8 Fundación ARAID, Zaragoza, Spain

**Keywords:** Glycoconjugates, Molecular biophysics

## Abstract

Core-fucosylation is an essential biological modification by which a fucose is transferred from GDP-β-L-fucose to the innermost N-acetylglucosamine residue of *N*-linked glycans. A single human enzyme α1,6-fucosyltransferase (FUT8) is the only enzyme responsible for this modification via the addition of an α-1,6-linked fucose to *N*-glycans. To date, the details of substrate recognition and catalysis by FUT8 remain unknown. Here, we report the crystal structure of FUT8 complexed with GDP and a biantennary complex *N*-glycan (**G0**), which provides insight into both substrate recognition and catalysis. FUT8 follows an S_N_2 mechanism and deploys a series of loops and an α-helix which all contribute in forming the binding site. An exosite, formed by one of these loops and an SH3 domain, is responsible for the recognition of branched sugars, making contacts specifically to the α1,3 arm GlcNAc, a feature required for catalysis. This information serves as a framework for inhibitor design, and helps to assess its potential as a therapeutic target.

## Abstract (web summary)

Core-fucosylation of the *N*-glycan core is an essential biological modification and the α1,6- fucosyltransferase FUT8 is the only enzyme in humans that catalyses this modification through the addition of an α-1,6-linked fucose to *N*-glycans. Here the authors provide insights into FUT8 substrate recognition by determining the 1.95 Å crystal structure of human FUT8 complexed with GDP and a biantennary complex *N*-glycan.

## Introduction

Core-fucosylation is an essential biological modification of the *N*-glycan core in eukaryotes (except for plants and fungi) that is performed by the inverting α1,6-fucosyltransferase (FUT8)^1^. It is the most common type of fucose modification and occurs in the Golgi apparatus. FUT8 transfers a n l-fucose residue from GDP-β-l-fucose (GDP-Fuc) onto the innermost GlcNAc of *N*-glycan to form an α1,6-linkage^2^. Gene knockout of *FUT8* in mice led to early postnatal death, severe growth retardation and emphysema-like changes in the lung, and revealed that this modification is crucial for the activation of growth factor receptors^1,3^. FUT8 is also involved in cell adhesion and cell migration processes by influencing turnover and expression levels of E-cadherin^4^ and the activity of α3/β1 integrin^5^. Core-fucose also effects *N*-glycan conformation by favoring an extended orientation of the α1,6 arm^6^. This can lead to subtle changes in glycan recognition by lectin receptors^7^. However, core-fucosylation is likely most known for its capacity to adjust the humoral immune response by altering antibody-dependent cellular cytotoxicity (ADCC). Thus, different strategies for reducing core-fucosylation in therapeutic monoclonal antibodies have been developed to enhance ADCC^8^. Notably, FUT8 is up-regulated in numerous types of cancer, suggesting that blocking its activity could be a promising strategy for improving anti-tumor immune responses^9^.

The acceptor specificity of FUT8 requires the presence of a terminal GlcNAc moiety on the α1,3 arm of the *N*-glycan but shows a higher degree of flexibility on the α1,6 arm^10^. This substrate preference does not necessarily demand the presence of a peptide/protein^10^. On the contrary, FUT8 also fucosylates high mannose *N*-glycans lacking a terminal GlcNAc moiety on the α1,3 arm. However, in these cases a peptide/protein moiety attached to the first GlcNAc via an *N*-glycosidic linkage^11^ is necessary.

The crystal structure of the human FUT8 (*Hs*FUT8) apo form has been previously reported revealing that FUT8 is composed of a multi-domain enzyme that contains an *N*-terminal coiled-coil domain, a catalytic domain, which adopts a GT-B fold, and a *C*-terminal SH3 domain^12^. However, the lack of solved ternary complexes has impeded to obtain mechanistic insights into the glycosyl transfer reaction or reveal the molecular basis of the requirement for a terminal GlcNAc moiety on the α1,3 arm for optimal catalysis. Here, we have captured a ternary complex formed between the human FUT8 (*Hs*FUT8), GDP and the biantennary complex *N*-glycan **G0 (**see below) by X-ray crystallography, uncovering the molecular basis of FUT8 catalysis and recognition of acceptor substrates.

## Results

### Core-fucosylation of synthetic *N*-glycans by *Hs*FUT8

**Fig. 1:**
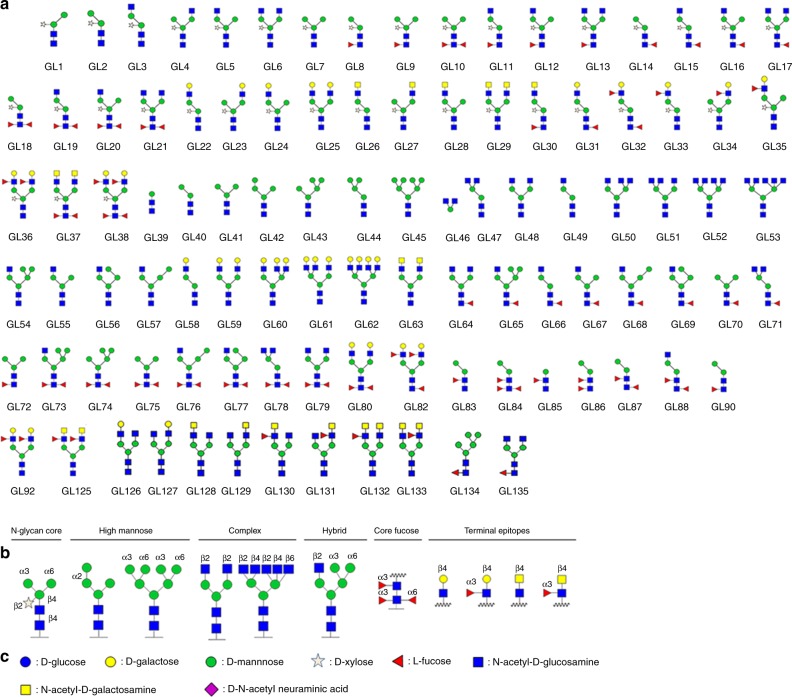
Glycan microarray employed for assessing *Hs*FUT8 substrate selectivity. **a** Pictogram representation of *N*-glycan structures included on the glycan array. **b** Specification of glycosidic bond configurations. **c** Monosaccharide symbol nomenclature according to Consortium of Functional Glycomics recommendations. See the following link for further information, http://www.functionalglycomics.org/static/consortium/Nomenclature.shtml.

**Fig. 2:**
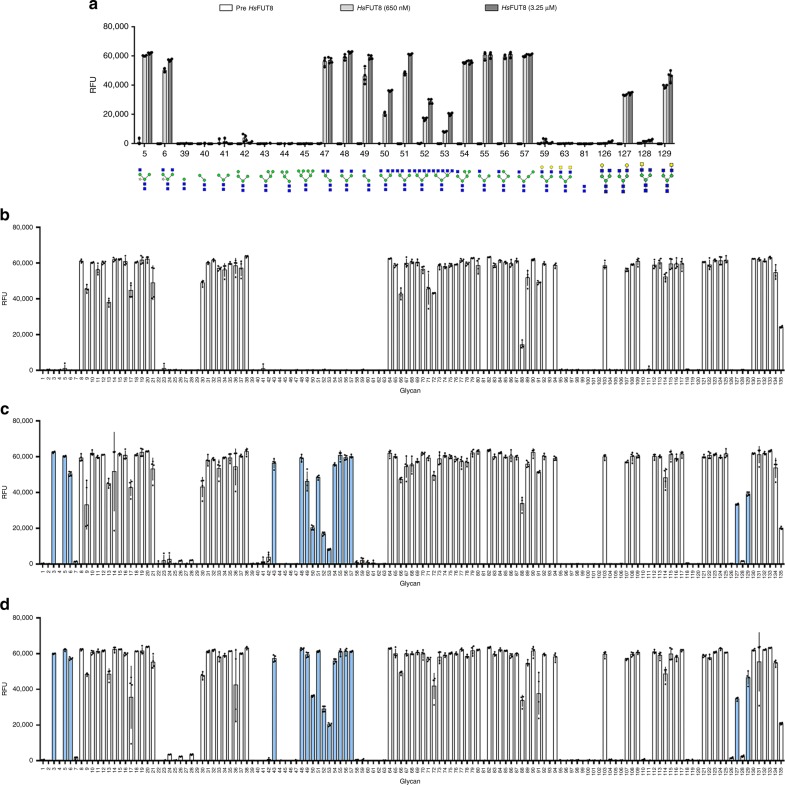
Activity assay of *Hs*FUT8 on glycan microarray. **a** *Aleuria aurantia* lectin (AAL-555) binding before and after incubation with recombinant *Hs*FUT8 at two different concentrations (650 nM and 3.5 μM) on a selection of glycan structures. **b** AAL-555-binding profile on a full glycan array before treatment with recombinant *Hs*FUT8. **c** AAL-555-binding assay after treatment with recombinant *Hs*FUT8 at 650 nM enzyme concentration and **d** AAL-555-binding assay after treatment with recombinant *Hs*FUT8 at 3.25 μM enzyme concentration. Histogram bars show the average fluorescence RFU (relative fluorescence units) values for four replicate spots on the array. Error bars represent the standard deviation of the average RFU values on the same microarray. *N*-glycans that show binding towards AAL after incubation with *Hs*FUT8 are highlighted in blue.

*Hs*FUT8 was successfully cloned, expressed and purified to homogeneity (Methods) to enable functional and structural characterization. The activity of *Hs*FUT8 on 5-aminopentyl **G0** was monitored by mass spectrometry that showed a mass increase of 146 Da corresponding to the incorporation of a fucose moiety into the glycan in the presence of GDP-Fuc (Supplementary Fig. 1). Then, we evaluated the *Hs*FUT8 acceptor substrate specificity on a microarray of synthetic *N*-glycans by expanding an earlier array version used by us in the study of *C. elegans* FUT8 (*Ce*FUT8)^13,14^ (Fig. 1, Supplementary Table 1**)**. The *Hs*FUT8 showed identical substrate acceptor preferences to the reported ones for *Ce*FUT8 (Fig. 2), and verified prior reports describing the importance of a terminal GlcNAc moiety in the α1,3 arm as a requirement for catalysis and a great promiscuity towards modifications on the α1,6 arm^10^.

### Architecture of the *Hs*FUT8-GDP-G0 complex

**Fig. 3:**
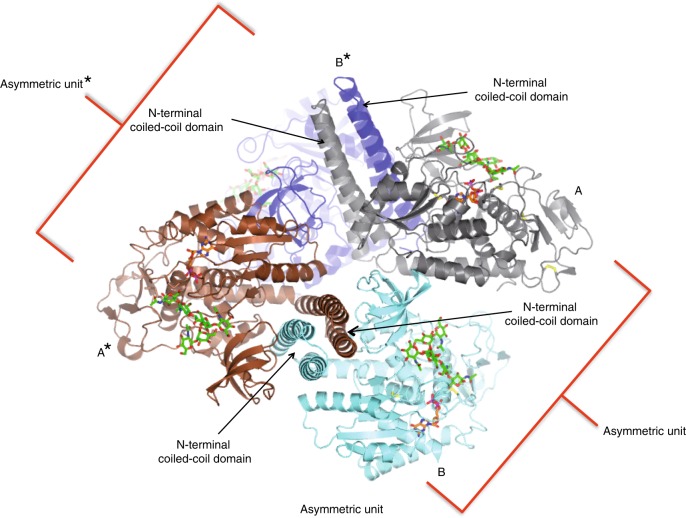
Dimeric structure of *Hs*FUT8. One *Hs*FUT8 molecule (A in gray) from one asymmetric unit forms a dimeric structure with another molecule (B* in blue) from the other asymmetric unit* through their corresponding coiled-coil domains.

**Fig. 4:**
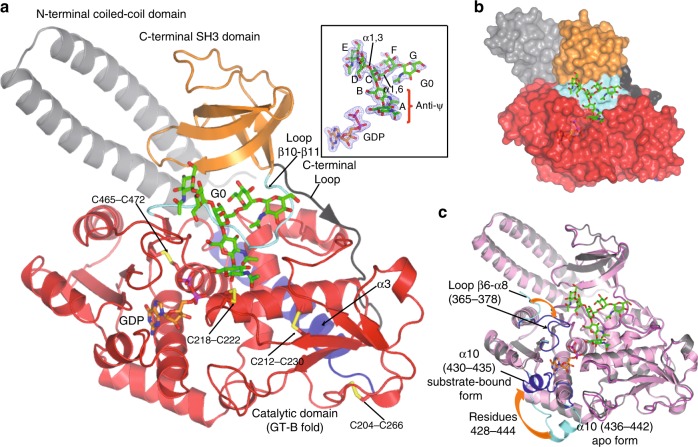
Overall structure of *Hs*FUT8 complexed to GDP and G0. **a** Ribbon structure of *Hs*FUT8 with GDP (orange carbon atoms) and **G0** (green carbon atoms). The coiled-coil, catalytic and SH3 domains are colored in gray, red and orange, respectively. The interdomain α3 and loop β10–β11 are colored in blue and aquamarine, respectively. Disulfide bridges are indicated as yellow sulfur atoms. The *C*-terminal loop is colored in black. (inset) Electron density maps are *F*_O_–*F*_C_ (blue) contoured at 2.2*σ* for GDP and **G0**. The labels of the sugar units (**A**–**G**) are also shown. **b** Surface representation of the *Hs*FUT8-GDP-**G0** complex. **c** Superposition of *Hs*FUT8 apo (pink) and substrate-bound (gray) forms. This figure also depicts the conformational changes visualized in residues 365–378 and 428–444. These regions are represented as aquamarine (apo form) and blue (substrate-bound form).

Further, we successfully obtained C2 crystals of *Hs*FUT8 in a complex with GDP and **G0** glycan (Table 1). The resulting crystals allowed us to solve the structure at high resolution (1.95 Å) and interpret the density map (Table 1). The asymmetric unit (AU) of C2 crystals contains two molecules of *Hs*FUT8 that are arranged as dimers with two molecules from the neighboring AU by direct contact between their corresponding *N*-terminal coiled-coil domains (Fig. 3). The root mean square deviation (RMSD) between both molecules belonging to chain A and B in the AU is 0.44 Å on 452 equivalent Cα atoms (Fig. 4; hereafter we will only discuss molecule A because it does not contain any disordered regions). As expected, the analysis of the *Hs*FUT8 structure with the DALI server^15^ revealed structural homology to two fucosyltransferases, namely the *Arabidopsis thaliana* FUT1 (*At*FUT1; PDB entries 5KX6, 5KOP, 5KOR, 5KWK and 5KOE^16,17^) and the human and *C. elegans* PoFUT2 (*Hs*PoFUT2 and *Ce*PoFUT2; PDB entries 4AP5, 4AP6 and 5FOE^18–20^). Although FUT8 is very distant to *At*FUT1 and *Hs*PoFUT2/*Ce*PoFUT2 in terms of acceptor substrates, the server rendered good scores implying that they superimposed fairly well (RMSDs of ~3.2 and ~5.0 Å between *At*FUT1 and *Hs*FUT8, and *At*FUT1 and *Hs*PoFUT2/*Ce*PoFUT2 crystal structures, respectively; the superimposed residues ranged from 429 to 459 residues).

**Table 1:** Data collection and refinement statistics.

	*HsFUT8* in complex with GDP and G0
**Data collection**	
Space group	C2
Cell dimensions	
* a*, *b*, *c* (Å)	208.04, 68.59, 173.92
*α*, *β*, *γ* (°)	90, 149.90, 90
Resolution (Å)	20–1.95 (2.06–1.95)^a^
*R*_merge_	0.133 (1.368)
*I*/σ*I*	6.4 (1.4)
Completeness (%)	99.9 (100)
Redundancy	4.4 (4.5)
**Refinement**	
Resolution (Å)	1.95
No. of reflections	394,687
*R*_work_/*R*_free_	0.185/0.218
No. of atoms	
Protein	7511
GDP	56
** G0**	180
Waters	506
Glycerol	102
*B*-factors (Å^2^)	
Protein	38.11
GDP	47.64
** G0**	44.70
Waters	49.00
Glycerol	63.01
R.m.s. deviations	
Bond lengths (Å)	0.0159
Bond angles (°)	2.0646

One crystal was used to determine the crystal structure. ^a^Values in parentheses are for highest-resolution shell.

The structure of *Hs*FUT8 complexed to GDP and **G0** depicts the three aforementioned domains and how they are connected with each other. While the catalytic domain is connected to the coiled-coil domain by α3, the SH3 domain is connected to the catalytic domain by the loop β10–β11 (Fig. 4a, Supplementary Fig. 2). While in general, densities for GDP and the rest of the **G0** molecule are very well defined, we only observe partial density for the GlcNAc moiety on the α1,6 arm (G^**G0**^). Of special note is the *anti*-*ψ* conformation for the core-chitobiose GlcNAc moieties (glycosidic linkage involving units A^**G0**^–B^**G0**^) of the *N*-glycan in the bound state (see below). This conformer is less stable relative to the typical *syn*-*ψ* conformation found in solution^21^ (see inset in Fig. 4a). In addition, the Manα1-6Man linkage of **G0** adopts an ‘extended *gg’* conformer^22^, with *ψ* and *ω* values of 174.7° and 50.4°, respectively. The structure also reveals that GDP is partly buried and located exclusively in the catalytic domain, while the inner part and the α1,3/α1,6 arms of **G0** are located in the catalytic domain and the exosite formed by the loop β10–β11 and SH3 domain, respectively (Fig. 4b). The presence of exosites for intimate binding to *N*-glycans was also observed in distant glycosyltransferases (GTs) and glycosyl hydrolases such as MGAT2 and MAN2A1, respectively^23^. Our structure also exhibits two striking conformational changes relative to the apo form (RMSD of 0.56 Å on 436 equivalent Cαs; Fig. 4c). Residues 428–444 (formed by a loop and α10) experience a large conformational change (the largest distance, close to 22 Å, was observed for Gly437 in both forms) that allows the partial closure of the GDP-Fuc-binding site and which protects the more hydrophobic part of the molecule (the guanosine moiety) from the solvent. Significantly, during the transition from the apo to the substrate-bound forms, a rearrangement of the α10 takes place in which α10^apo-form^ (residue 436–442) becomes unstructured and another α10, comprising residues 430–435, is formed. Furthermore, the loop β6–α8 (residues 365–378) is partly disordered in the apo form and suffers a conformational change in the presence of both ligands, leading to an ordered loop that contributes to the formation of both the GDP-Fuc and the acceptor binding sites (Fig. 4c).

### The sugar nucleotide-binding site

**Fig. 5:**
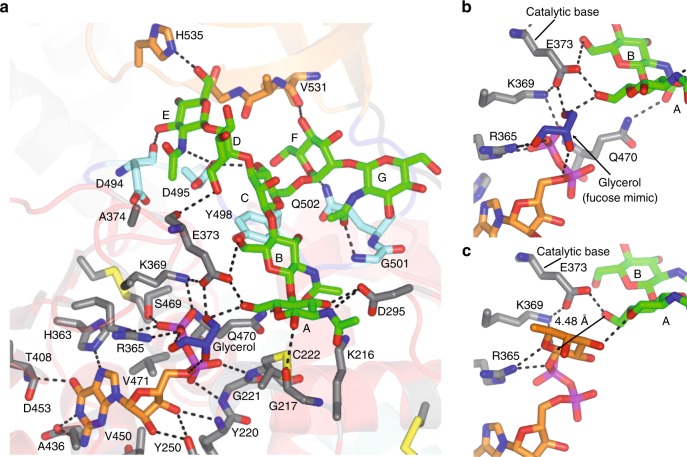
Structural features of GDP-Fuc and G0 binding sites. **a** Complete GDP-Fuc and **G0** binding sites of the *Hs*FUT8-GDP-**G0** complex. The residues forming the GDP-Fuc/A^**G0**^/B^**G0**^ and C^**G0**^/D^**G0**^/E^**G0**^/F^**G0**^/G^**G0**^ binding sites are depicted as gray and aquamarine/orange carbon atoms, respectively. GDP, **G0** and glycerol are shown as orange, green and blue carbon atoms, respectively. Hydrogen bond interactions are shown as dotted black lines. **b**, **c** Close-up view of the binding site region of the *Hs*FUT8-GDP-**G0** and *Hs*FUT8-GDP-Fuc-**G0** complexes showing the essential residues (Arg365, Lys369 and Glu373) that are major players in the plausible S_N_2 single-displacement reaction mechanism. Note the proximity and the orientation of the A^**G0**^ OH6 to the anomeric carbon (4.48 Å) which is compatible with the inversion of the configuration during the reaction.

The *Hs*FUT8-binding site is large and set up by the GDP-Fuc and the *N*-glycan binding sites (Fig. 5a, b). The guanine moiety of GDP establishes CH–π interactions with Ala436, Val450 and Val471 while the guanosine moiety is tethered via hydrogen bonds to Tyr250/His363/Asp453 side chains and Tyr220/Thr408 backbones. The pyrophosphate interacts with Gly221/Cys222/Gln470 backbones, Lys369/Arg365/Ser469 side chains and a glycerol moiety. The presence of positively charged residues like Arg365 and Lys369 is typically found in GT-B fold GTs, which do not bind to metal ions^24^. These residues replace the metal present in GT-A fold GTs and stabilize the pyrophosphate groups and the right conformations of sugar nucleotides for catalysis^24^. The glycerol moiety is a fucose mimic that occupies a similar position as found for fucose (see Methods, Fig. 5b, c, Supplementary Fig. 3), and further interacts with Arg365 and Glu373 side chains, and A^**G0**^ OH6, the site of fucosylation in **G0** (Fig. 5b). The GDP-Fuc coordinates were obtained from the crystal structure of *Ce*PoFUT1 complexed to GDP-Fuc (PDB entry: 3ZY6 (ref. ^25^)) and the resulting *Hs*FUT8-GDP-Fuc-**G0** complex was further minimized using molecular mechanics (MM) calculations, as shown in Methods. Two hydroxyl groups of the glycerol molecule occupy positions that mimic fucose OH4 and the endocyclic oxygen. In addition, one of these two hydroxyl groups is also found in interaction distance of Arg365 and mimics the conserved interaction observed by the fucose endocyclic oxygen and Arg365 (Fig. 5b, c, Supplementary Fig. 3). Glu373 side chain further interacts with A^**G0**^ OH6 and Lys369 side chain, which helps stabilize the negative charge of Glu373 carboxylate. This could improve the catalytic base character of Glu373 for deprotonating the A^**G0**^ OH6 (Fig. 5a, b). The importance of Arg365, Lys369 and Glu373 for catalysis was previously confirmed by Ala substitutions by site-directed mutagenesis of these residues, which rendered the resulting mutants fully inactive^12^. The location of Arg365, Lys369 and Glu373 in the mobile loop β6–α8 demonstrates the pivotal relevance of this loop in binding to the ligands and catalysis. Markedly, structure of *Hs*FUT8 with GDP-Fuc (see Methods) and **G0** could demonstrate the inversion at the anomeric carbon of fucose with respect to GDP-Fuc (Fig. 5c). Therefore, these results are compatible with an S_N_2 single-displacement reaction mechanism, which is deployed by most inverting GTs^24^.

### The *N*-glycan-binding site

Unlike the intimate recognition of GDP by *Hs*FUT8, **G0** displays less contacts with the protein (Fig. 5a), in line with previous SPR data in which the binding of GDP-Fuc was ~40-fold stronger than the binding of **G0** to the enzyme^26^ (Fig. 5a). A^**G0**^ is recognized through hydrogen bonds formed between A^**G0**^ OH1 and Gly217 backbone, A^**G0**^ acetamide NH and Asp295 side chain, and A^**G0**^ OH3 and Asp295/Lys216 side chains. B^**G0**^ is tethered via interactions formed between B^**G0**^ OH6 and Glu373 side chain, and B^**G0**^ acetamide carbonyl group and Gln470 side chain. The unusual *anti*-*ψ* conformation of A^**G0**^ and B^**G0**^, which is imposed by the enzyme, is a strict requirement for the A^**G0**^ OH6 fucosylation site facing GDP-Fuc (Fig. 5a). Catalysis can only take place in the *anti*-*ψ* conformation even though this conformation is less energetically favorable than the solution-phase *syn*-*ψ* conformation (−8.9 versus −9.5 kcal/mol, estimated by MM calculations for the disaccharide containing A^**G0**^–B^**G0**^ units).

**Fig. 6:**
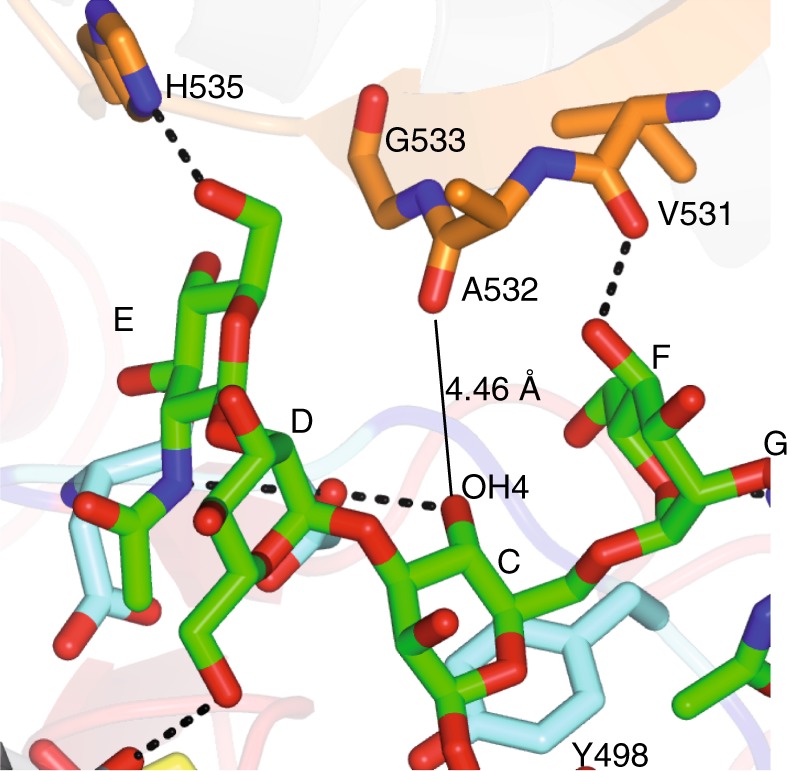
The bisecting GlcNAc in *N*-glycans. *N*-glycans carrying a bisecting GlcNAc are not substrates of *Hs*FUT8 likely due to a steric clash between the bisecting GlcNAc with Ala532 (distance of 4.46 Å between the Ala532 backbone and C^G0^ OH4). Note that the bisecting GlcNAc would be bound to C^G0^ via the C^G0^ OH4, and it is this GlcNAc who would have a steric clash with Ala352. It is also likely that the addition of the bisecting GlcNAc causes conformational changes to the neighboring branching sugar moieties, which could contribute to disrupt the binding to *Hs*FUT8.

The sugars forming the branching part of **G0** (C^**G0**^, D^**G0**^, E^**G0**^, F^**G0**^ and G^**G0**^) are located in the exosite formed by the loop β10–β11 and the SH3 domain (Fig. 5a). C^**G0**^ establishes a CH–π interaction with Tyr498 and provides one hydrogen bond between C^**G0**^ OH4 and Asp495 side chain. D^**G0**^ is poorly recognized by a lonely hydrogen bond between D^**G0**^ OH6 and the Glu373 backbone. However, the terminal GlcNAc moiety in the α1,3 arm, E^**G0**^, is the most intimately recognized monosaccharide residue of all branched sugars. E^**G0**^ established three hydrogen bonds that are formed between E^**G0**^ OH6 and His535 side chain, E^**G0**^ OH2 and Asp494 backbone, and E^**G0**^ acetamide NH and Asp495 side chain. In addition, the E^**G0**^ acetamide methyl group is engaged in a hydrophobic interaction with Ala374 (Fig. 5a). The contacts that E^**G0**^ establishes with the enzyme could explain the strict requirement for a GlcNAc moiety in this position. This unit improves the binding of the α1,3 arm, and in turn, provides stabilization of the entire molecule in the bound state. On the contrary, the branching sugars on the α1,6 arm are far less well recognized, which provides a plausible explanation for the larger promiscuity towards the α1,6-mannose branch^10^. The F^**G0**^ endocyclic oxygen and F^**G0**^ OH4 establish two hydrogen bonds with Gln502 side chain and Val531 backbone, respectively, while other potential interactions between G^**G0**^ and *Hs*FUT8 (G^**G0**^ carbonyl group and G^**G0**^ OH3 with Gly501 and Gln502 backbones, respectively) cannot be confirmed due to the less well-defined density in this sugar residue (Figs. 4a and 5a). The addition of a bisecting GlcNAc in **G0** would preclude fucosylation by FUT8 because this residue is likely to impose a significant steric hindrance to the complex (Fig. 6), explaining why *N*-glycans with bisecting GlcNAc are not substrates of FUT8 (ref. ^27^).

## Discussion

**Fig. 7:**
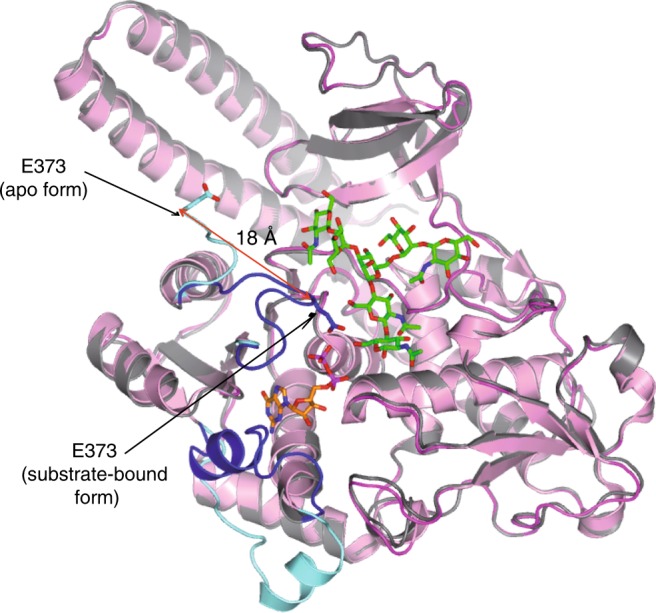
Superposition of *Hs*FUT8 apo (pink) and substrate-bound (gray) forms. This figure also depicts the conformational changes visualized in Glu373 (in aquamarine and blue for the apo and substrate-bound form, respectively).

FUT8 is the only enzyme responsible for core-fucosylation on mammalian *N*-glycoproteins. Despite many years of research on the enzymology and chemistry of FUT8, its reaction mechanism remained elusive and the requirement for a terminal GlcNAc moiety on the α1,3 arm of the *N*-glycan for optimal fucosylation was not well understood. The structure of the *Hs*FUT8 apo form provided the architecture of this enzyme, which is formed by three different domains^12^. However, this structure did not offer any clues for the reaction mechanism or substrate recognition. Here, we have determined the crystal structure of a functional *Hs*FUT8 in complex with GDP and **G0**. Intriguingly, the structure reveals that residues 436–442, in which one loop and α10 are present, and the loop β6–α8 suffer large conformational changes in the presence of the ligands forming the GDP-Fuc and acceptor binding sites. Though conformational changes of loops have been visualized in inverting and retaining GTs^24,28,29^, the motion here of the loop β6–α8 in the presence of ligands is unique because this loop brings in key residues that recognize both GDP and **G0**. In particular, Arg365, Lys369 and Glu373 contribute not only to the binding but also to catalysis, with Glu373 acting as the catalytic base for A^**G0**^ OH6. This is compatible with an S_N_2 single-displacement reaction mechanism. Generally, GT-A or GT-B inverting GTs following an S_N_2 inverting mechanism contain an amino acid acting as a catalytic base^24^ (typically Glu, Asp, or His). However, in most structures, these residues acting as catalytic residues are already located in the binding site in the absence of ligands^24^. Hence and to our knowledge, FUT8 exemplifies a unique case in which the catalytic base is brought to the binding site in the presence of ligands (note that the distance of Glu373 Cαs of the apo and substrate-bound form is ca. 18 Å; Fig. 7).

Previously and based on computational studies, *Hs*FUT8 was suggested to follow an S_N_2 mechanism in which the β-phosphate was acting as the catalytic base^26^. Similarly, the β-phosphate acting as the catalytic base was proposed for the inverting *Ce*PoFUT1^25^, though in this case an S_N_1 mechanism was suggested. In both enzymes, there was no basic residue present in the active site that could act as an assisting base. However, and based on our crystal structure, it is now clear that the authors could not predict the motion of the loop β6–α8, which brings the essential catalytic base Glu373. This demonstrates that it is imperative to obtain ternary complexes for these enzymes to elucidate their catalytic mechanism. Our S_N_2 inverting mechanism is also reinforced by the previous elucidation of ternary complexes for the closest structural homologs, *At*FUT1 (ref. ^17^) and *Ce*PoFUT2 (ref. ^18^). For both enzymes, residues such as Asp and Glu, respectively, were also acting as the catalytic bases.

At the level of the *N*-glycan-binding site, four major structural features are responsible of the selectivity and recognition of *Hs*FUT8 on *N*-glycans: (a) the less stable *anti*-*ψ* conformation for the core-chitobiose GlcNAc moieties of the *N*-glycan in the bound state is a pre-requisite for core-fucosylation, since it provides the A^**G0**^ OH6 in close contact to GDP-Fuc; (b) the CH–π interaction between Tyr498 and C^**G0**^ is likely crucial for a right orientation of the branching sugars; (c) the existence of an exosite formed by the loop β10–β11 and SH3 domain is responsible for branching sugar recognition and, in particular, for the α1,3 arm GlcNAc binding. SH3 domains are small protein domains of about 60 amino acid residues present in a large number of proteins and well-known for driving protein–protein interactions^30^. The fact that residues from the *Hs*FUT8 SH3 domain recognize sugar moieties implies the broad versatility of these domains in recognition of different molecules; and (d) the most intimately recognition for the α1,3 arm GlcNAc explains why FUT8 requires the presence of a terminal GlcNAc moiety on the α1,3 arm of the *N*-glycan for optimal fucosylation. This ligand-binding model also explains our own data using *Hs*FUT8 on a microarray of synthetic *N*-glycans and prior reports^10,13,14^. However, our structure does not offer clues on how FUT8 fucosylates less optimally high mannose *N*-glycans linked to glycoproteins and peptides that lack a terminal GlcNAc on the α1,3-branch and which will require further structural studies.

In conclusion, the structure reported here shows how FUT8 recognizes *N*-glycans and provides information on the enzymatic mechanism. Our structure may assist in the development of inhibitors with a potential value for cancer therapy or as an alternative for the production of non-fucosylated antibodies with enhanced ADCC.

## Methods

### Glycan preparation

For the preparation of the biantennary complex A2 *N*-glycan (named here as **G0**), sialyl glycopeptide (SGP) predominantly glycosylated with A2G2S2 glycan was isolated from hen's egg yolk according to a published procedure^31^ and further enzymatically and chemically processed. Treatment of the glycopeptide with acetic acid at 80 °C removed sialic acid residues^32^ and deglycosylation  with PNGase F (Asparia Glycomics) produced a mix of mono and bis-galactosylated glycans^33^. The obtained mixture (110 mg, 66.8 µmol) was dissolved in 50 mM citric phosphate buffer (8 mL, pH = 5). *Aspergillus niger* β-galactosidase (8.9 U/mg, 14.2 mg, in 1260 µL buffer) and an aqueous solution of 0.02% NaN_3_ were added. The reaction mixture was incubated at 37 °C and the reaction progress monitored via MALDI-TOF MS. After complete degalactosylation, MeOH (5 mL) was added and the reaction mixture centrifuged at 4500 r.p.m. MeOH was removed under reduced pressure and the supernatant was loaded onto a Bond elute graphite cartridge (10 g) and eluted with H_2_O (250 mL), 25% MeOH/H_2_O (500 mL), 50% MeOH/H_2_O (250 mL). The collected fractions of 50% MeOH were analyzed by MALDI-TOF MS and fractions containing pure product were pooled and lyophilized. The pure product **G0** was obtained as a white solid (69.1 mg, 52.5 µmol, 79%). ^**1**^**H NMR** (500 MHz, D_2_O, 298 K): δ 5.10 (s, 1H, αH-1_GlcNAc-1_), 5.02 (s, 1H, H-1_αMan-2_), 4.83 (s, 1H, H-1_αMan-3_), 4.68 (s, 1H, H-1_βMan-1_), 4.62–4.59 (m, 1H, βH-1_GlcNAc-1_), 4.54–4.50 (m, 1H, H-1_βGlcNAc-2_), 4.46 (d, *J* = 8.4 Hz, 2H, H-1_βGlcNAc-3_, H-1_βGlcNAc-4_), 4.16 (s, 1H, H-2_αMan-2_), 4.10 (s, 1H, H-2_αMan-3_), 4.02 (s, 1H, H-2_αMan-1_), 3.90–3.31 (m, 39H), 1.99 (s, 3H, CH_3,GlcNAc_), 1.96 (s, 6H, CH_3,GlcNAc_), 1.95 (s, 3H, CH_3,GlcNAc_). ^**13**^**C NMR** (126 MHz, D_2_O, 298 K): δ 174.8, 174.7, 174.6 (4× CO), 101.3 (C1_βGlcNAc-2_), 100.4 (C1_βMan-1_), 99.5 (C1_βMan-2_, C1_βGlcNAc2_, C1_βGlcNAc3_), 97.0 (C1_βMan-3_), 94.8 (βC1_GlcNAc-1_), 90.6 (αC1_αGlcNAc-1_), 79.6, 79.5, 79.2, 76.4 (C2_Man-3_), 76.3 (C2_Man-1_), 75.8, 74.3, 73.5, 73.4, 73.2, 72.8, 72.4, 72.0, 69.9 (C2_Man-2_), 69.2, 67.3, 65.7, 61.6, 60.6, 59.9, 55.3 (C2_GlcNAc-4_), 54.9 (C2_GlcNAc-3_), 53.6 (C2_GlcNAc-1_, C2_GlcNAc-2_), 22.3, 22.2, 21.8 (4× CH_3_). **HRMS** (MALDI-TOF): calcd for C_50_H_84_N_4_O_36_Na [M + Na]^+^ 1339.475, found 1339.515.

### Activity assay on 5-aminopentyl G0

**G0** containing a C5 linker at the anomeric position of the reducing end (5-aminopentyl **G0**^34^) (1 nmol), guanosine 5′-diphospho-β-L-fucose (GDP-Fuc) (2 nmol) and *Hs*FUT8 (18 µM) in Tris 25 mM, 150 mM NaCl pH = 7.5 were incubated at room temperature (rt) for 5 h. MALDI-TOF MS analysis of the reaction mixture employing a solution of 2,5-dihydroxybenzoic acid (DHB, 5 mg/mL in acetonitrile) as matrix showed the formation of fucosylated 5-aminopentyl **G0**. Control experiments lacking *Hs*FUT8 and GDP-Fuc were performed showing the unaltered 5-aminopentyl **G0** by MALDI-TOF MS (Supplementary Fig. 1).

### Glycan array analysis

*Hs*FUT8 screening on glycan microarrays was performed as previously described with slight modifications^13^. Each subarray was incubated with a solution (200 µL) containing two different concentrations of FUT8 (650 nM and 3.25 µM), GDP-Fuc (0.5 mM), MnCl_2_ (10 mM) in Tris 25 mM, 150 mM NaCl pH = 7.5 at rt overnight. The supernatant was removed and the slide was washed with PBS and water. The introduction of Fuc residues by the action of FUT8 was probed by incubation with *Aleuria aurantia* lectin (Vector laboratories, Burlingame, CA, USA) labeled with Alexa Fluor™ 555 NHS succinimidyl ester (Thermo Fisher, AAL-555, 60 µg/mL in binding buffer: Tris 25 mM, 150 mM NaCl pH = 7.5 containing 0.01% Tween-20) at rt for 1 h. The microarray slide was washed with binding buffer, and water and fluorescence was analyzed on an Agilent G265BA microarray scanner system (Agilent Technologies, Santa Clara, USA). Quantification was performed with ProScanArray® Express (Perkin Elmer, Shelton, USA) and Microsoft Excel software. The average of mean RFU values after background subtraction and standard deviation for four replicate spots was represented as histograms employing GraphPad Prism 6 software.

### Expression and purification of *Hs*Fut8

The DNA sequence encoding amino acid residues 68–575 of the *Hs*FUT8 was codon optimized and synthesized by GenScript (USA) for expression in HEK293 cells (see Supplementary Table 2 for the codon optimized sequence). The DNA, containing at the 5′-end a recognition sequence for *Kpn*I, and at the 3′ end a stop codon and a recognition sequence for *Xho*I, was cloned into a modified pHLSec containing after the secretion signal sequence a 12xHis tag, a superfolder GFP^35^ and a Tobacco Etch Virus (TEV) cleavage site, rendering the vector pHLSec-12His-*GFP*-TEV-*HsFUT8*. Both the synthesis of the *HsFUT8* construct and the engineered pHLSec together with the cloning of *HsFUT8* into pHLSec-12His-*GFP*-TEV were performed by GenScript.

pHLSec-12His-*GFP*-TEV-*HsFUT8* was transfected into HEK293F cell line (Thermo Fisher Scientific) as described below. Cells were grown in suspension in a humidified 37 °C and 8% CO_2_ incubator with rotation at 125 r.p.m. Transfection was performed at a cell density of 2.5 × 10^6^ cell/mL in fresh media F17 serum-free media with 2% Glutamax and 0.1% P188. For each 150 mL of culture, 450 μg of the plasmid (1 μg/μL) was diluted to 135 µL with sterilized 1.5 M NaCl. This mixture was added to each 150 mL cell culture flask and incubated for 5 min in the incubator. After that, 1.35 mg of PEI-MAX (1 mg/mL) was mixed to 135 µL with sterilized 1.5 M NaCl and added to the cell culture flask. Cells were diluted 1:1 with pre-warmed media supplemented with valproic acid 24 h post-transfection to a final concentration of 2.2 mM. Cells were harvested 6 days post-transfection by spinning down at 300 × *g* for 5 min, after which the supernatants were collected and centrifuged at 4,000 × *g* for 15 min.

Supernatant was dialyzed against buffer A (25 mM TRIS pH 7.5, 300 mM NaCl) and loaded into a His-Trap Column (GE Healthcare). Protein was eluted with an imidazol gradient in buffer A from 10 mM up to 500 mM. Buffer exchange to 25 mM TRIS pH 7.5, 150 mM NaCl (buffer B) was carried out using a HiPrep 26/10 Desalting Column (GE Healthcare). TEV protease was then added in a ratio 1:50 (TEV:protein) to the fusion construct in order to cleavage the His-GFP. After 20 h of reaction at 18 °C, the cleavage was satisfactorily verified through SDS-PAGE.

TEV protease and GFP were later removed from the solution using a His-Trap Column (GE Healthcare), and isolated *Hs*FUT8 was then loaded into a HiLoad 26/60 Superdex 75 Colum (GE Healthcare), previously equilibrated with buffer B. Quantification of protein was carried out by absorbance at 280 nm using his theoretical extinction coefficient (*ε*_280 nm_^*Hs*Fut8^ = 90,760 M^−1^ cm^−1^).

### Crystallization and data collection

Crystals of the *Hs*FUT8 were grown by sitting drop experiments at 18 °C by mixing 0.5 μL of protein solution (4.5 mg/mL *Hs*FUT8, 5 mM GDP and 5 mM **G0** in buffer B) with an equal volume of a reservoir solution (0.1 M carboxilic acids, 0.1 M buffer system 3 pH 8.5 and 30% precipitant mix 1 (Molecular Dimensions). The crystals were cryoprotected in mother liquor containing 30% glycerol and flash frozen in liquid nitrogen.

### Structure determination and refinement

Diffraction data were collected on the synchrotron beamline I24 of the Diamond Light Source (Harwell Science and Innovation Campus, Oxfordshire, UK) at a wavelength of 0.97 Å and a temperature of 100 K. Data were processed and scaled using XDS^36^ and CCP4 (refs. ^37,38^) software packages. Relevant statistics are given in Table 1. The crystal structure was solved by molecular replacement with Phaser^37,38^ using the PDB entry 2DE0 as the template. Initial phases were further improved by cycles of manual model building in Coot^39^ and refinement with REFMAC5 (ref. ^40^). Further rounds of Coot and refinement with REFMAC5 were performed to obtain the final structure. The final model was validated with PROCHECK; model statistics are given in Table 1. The AU of the C2 crystal contained two molecules of *Hs*FUT8. The Ramachandran plots for the *HsFUT8*-GDP-**G0** show that 92.1%, 7.6%, 0.2% and 0% of the amino acids are in most favored, allowed, generously allowed and disallowed regions, respectively.

### Molecular mechanics minimizations

GDP-Fuc was obtained from *Ce*PoFUT1 in complex with GDP-Fuc (PDB entry: 3ZY6 (ref. ^25^)) and then it was superimposed on our crystal structure. The calculations were carried out on the complexes using AMBER 18 package, which was implemented with ff14SB, GLYCAM06 and GAFF force fields. The complex was immersed in a water box with a 10 Å buffer of TIP3P water molecules and neutralized by adding explicit Na^+^. A two-stage geometry optimization approach was performed with a total of 5000 minimization steps and using the default settings of AMBER 18. The first stage minimizes only the positions of solvent molecules and ions, and the second stage is an unrestrained minimization of all the atoms in the system.

### Reporting summary

Further information on research design is available in the Nature Research Reporting Summary linked to this article.

## Supplementary information

Supplementary Information

Peer Review File

Reporting Summary

## Data Availability

The crystal structure of the *Hs*FUT8-GDP-**G0** complex was deposited at the RCSB PDB with accession code 6TKV. The source data underlying Fig. 2 are provided as a Source Data file. Other data are available from the corresponding author upon reasonable request.

## Source Data

Source Data
