# Supplementary material for: Structural basis for substrate specificity and catalysis of α1,6-fucosyltransferase
Source: Nat Commun. 2020 Feb 20;11:973. doi: 10.1038/s41467-020-14794-z (PMC7033129; doi:10.1038/s41467-020-14794-z)
Supplement: Supplementary file 1 — Supplementary Information [file 41467_2020_14794_MOESM1_ESM.pdf]

Supplementary Information

Structural basis for substrate specificity and catalysis of α1,6-fucosyltransferase

Ana García-García, Laura Ceballos-Laita, et al.

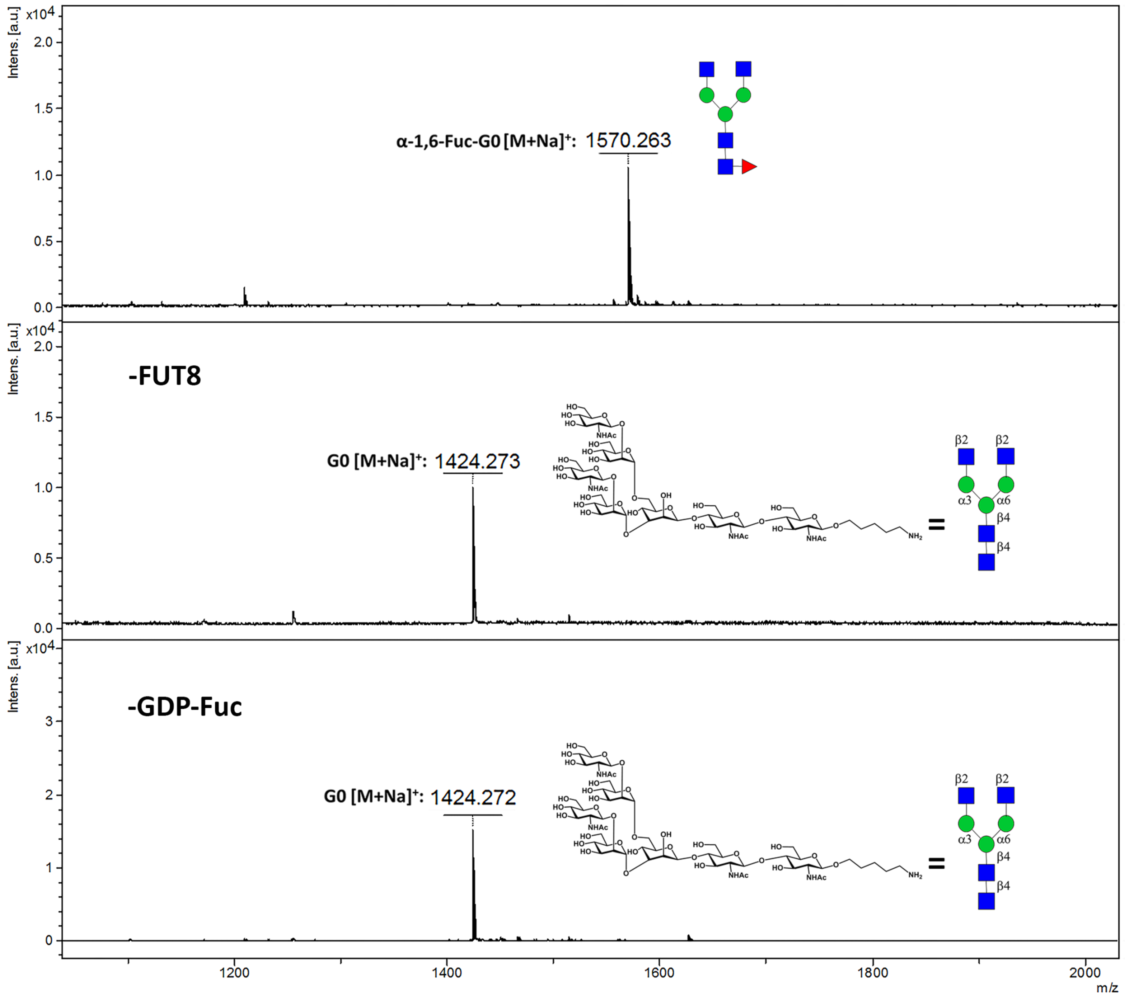

Supplementary Figure 1. **Activity of recombinant *Hs*FUT8 on 5-aminopentyl G0.** MALDI-TOF spectrum showing the introduction of fucose on 5-aminopentyl glycan **G0** by the action of recombinant *Hs*FUT8 (top). Control experiments lacking recombinant *Hs*FUT8 (middle) and without GDP-Fuc (bottom) showing the presence of unaltered 5-aminopentyl **G0**. These experiments were performed in duplicate.

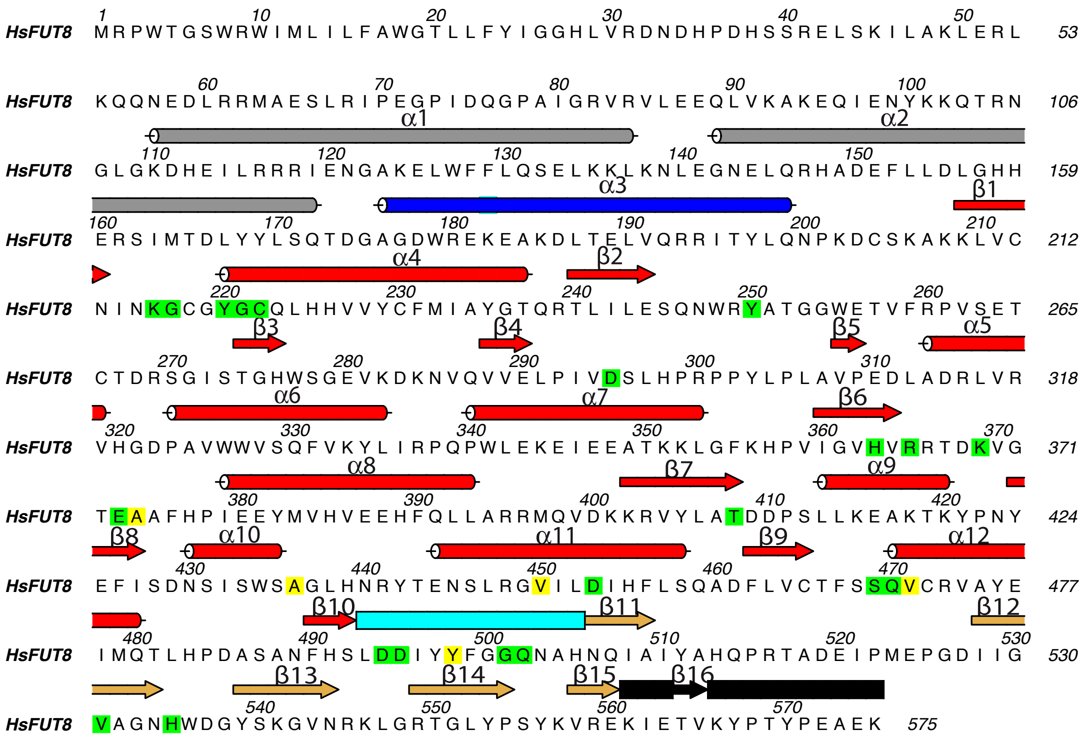

Supplementary Figure 2. Sequence of *Hs*FUT8 depicting the different domains. Secondary structure elements are shown above the sequence (α-helices and β-strands). The secondary structure elements are colored in grey, red and orange for the coiled-coil domain, the catalytic domain and the SH3 domain, respectively. The α3 that connects the coiled-coil domain and the catalytic domain is colored in blue. The loop β10-β11 (indicated as a rectangle) that connects the catalytic domain with the SH3 domain is colored in aquamarine. The C-terminal loop (indicated as a rectangle) and the β16 are colored in black. Residues interacting by hydrogen bonds with the ligands are highlighted in green while residues interacting by hydrophobic interactions with **G0** and GDP are highlighted in yellow.

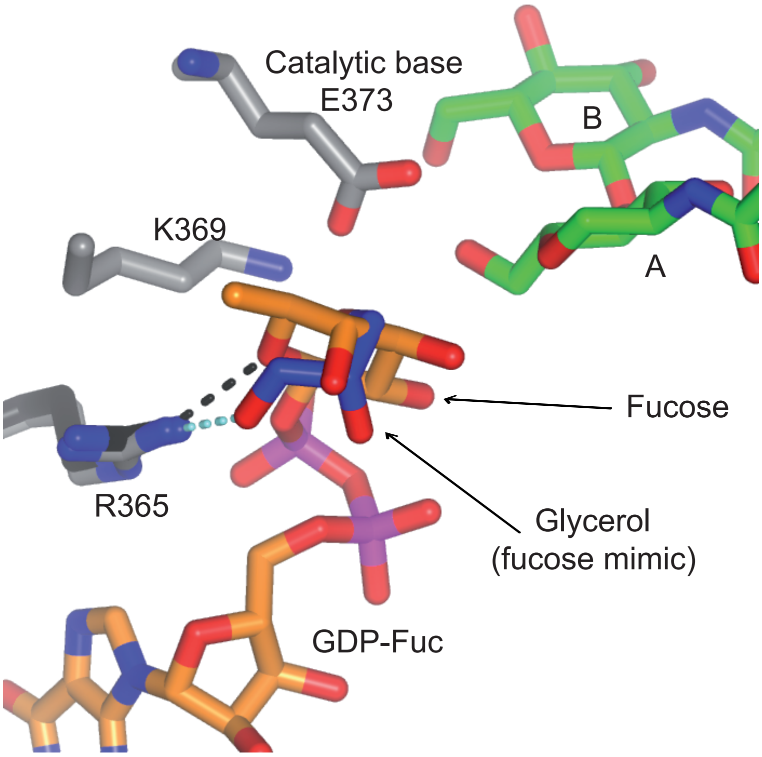

Supplementary Figure 3. Superposition of the *Hs*FUT8-GDP-G0 and *Hs*FUT8-GDP-Fuc-G0 structures. GDP-Fuc, **G0** and glycerol are shown as orange, green and blue carbon atoms, respectively. *Hs*FUT8-GDP-Fuc-**G0** complex was derived from molecular mechanics calculations. GDP-Fuc coordinates were obtained from the crystal structure of *Ce*PoFUT1 complexed to GDP-Fuc (PDB entry: 3ZY6) and the resulting complex was further minimized as described in Methods. Note that GDP from the *Hs*FUT8-GDP-**G0** complex is not shown for illustration purposes. Only the conserved hydrogen bonds conserved between fucose/glycerol and Arg365 are shown. Hydrogen bond interactions are shown as dotted black and aquamarine lines for GDP-Fuc and Arg365, and glycerol and Arg365, respectively.

Supplementary Table 1. Additional glycan structures included on microarrays.

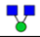	**GL46**	GlcNAcβ1-2(GlcNAcβ1-4)Manα-sp	
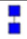	**GL81**	GlcNAcβ1-4GlcNAcβ-sp	
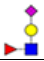	**GL89**	Neu5Acα2-3Galβ1-4(Fucα1-3)GlcNAcβ-sp	SialylLeX
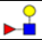	**GL91**	Galβ1-4(Fucα1-3)GlcNAcβ-sp	LeX
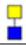	**GL93**	GalNAcβ1-4GlcNAcβ-sp	LDN
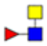	**GL94**	GalNAcβ1-4(Fucα1-3)GlcNAcβ-sp	
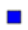	**GL95**	GlcNAcβ-sp	
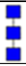	**GL96**	GlcNAcβ1-4GlcNAcβ1-4GlcNAcβ-sp	
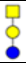	**GL97**	GalNAcβ1-4Galβ1-4GlcNAcβ-sp	GG3
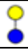	**GL98**	Galβ1-4Glcβsp	lactose
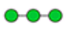	**GL99**	Manα1-2Manα1-2Manα-sp	
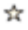	**GL100**	Xylβ-sp	
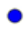	**GL101**	Glcβ-sp	
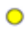	**GL102**	Galβ-sp	
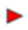	**GL103**	Fucα-sp	
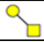	**GL104**	Gala1-3GalNAca-sp	
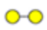	**GL105**	Galα1-2Galβ-sp	
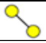	**GL106**	Galα1-3Galβ-sp	
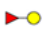	**GL107**	Fucα1-2Galβ-sp	
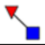	**GL108**	Fucα1-3GlcNAcβ-sp	
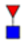	**GL109**	Fucα1-4GlcNAcβ-sp	
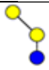	**GL110**	Galα1-3Galβ1-4Glcβ-sp4	
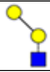	**GL111**	Galα1-3Galβ1-4GlcNAcβ-sp	
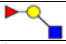	**GL112**	Fucα1-2Galβ1-3GlcNAcβ-sp	Le^{d} (H type 1)
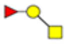	**GL113**	Fucα1-2Galβ1-3GalNAcα-sp	H (type 3)
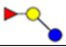	**GL114**	Fucα1-2Galβ1-4Glcβ-sp	H (type 6)
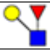	**GL115**	Galβ1-3(Fucα1-4)GlcNAcβ-sp	Le^{a}
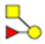	**GL116**	GalNAcα1-3(Fucα1-2)Galβ-sp	A_{tri}
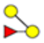	**GL117**	Galα1-3(Fucα1-2)Galβ-sp	B_{tri}
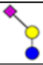	**GL118**	Neu5Acα2-3Galβ1-4Glcβ-sp	3´SL
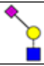	**GL119**	Neu5Acα2-3Galβ1-4GlcNAcβ-sp	3´SLN
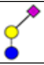	**GL120**	Neu5Acα2-6Galβ1-4GlcNAcβ-sp	6´SL
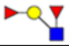	**GL121**	Fucα1-2Galβ1-3(Fucα1-4)GlcNAcβ-sp	Le^{b}
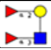	**GL122**	Fucα1-2Galβ1-3(Fucα1-3)GlcNAcβ-sp	Le^{y}
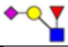	**GL123**	Neu5Acα2-3Galβ1-3(Fucα1-4)GlcNAcβ-sp	$SialLe^{a}$
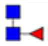	**GL124**	GlcNAcβ1-4(Fucα1-6)GlcNAcβ-sp	$FucGlcNAc_2$

Supplementary Table 2. DNA sequence of the codon optimized construct for expression of *Hs*FUT8. Note that the nucleotides sequences for the KpnI and XhoI endonucleases are highlighted in red and blue, respectively. The stop codon nucleotides are highlighted in green.

GGTACCCGGATACCAGAAGGCCCTATTGATCAGGGGCCAGCTATAGGAAGA
GTACGCGTTTTAGAAGAGCAGCTTGTTAAGGCCAAAGAACAGATTGAAAAT
TACAAGAAACAGACCAGAAATGGTCTGGGGAAGGATCATGAAATCCTGAG
GAGGAGGATTGAAAATGGAGCTAAAGAGCTCTGGTTTTTCCTACAGAGTGA
ATTGAAGAAATTAAAGAACTTAGAAGGAAATGAACTCCAAAGACATGCAG
ATGAATTTCTTTTGGATTTAGGACATCATGAAAGGTCTATAATGACGGATCT
ATACTACCTCAGTCAGACAGATGGAGCAGGTGATTGGCGGGAAAAAGAGG
CCAAAGATCTGACAGAACTGGTTCAGCGGAGAATAACATATCTTCAGAATC
CCAAGGACTGCAGCAAAGCCAAAAAGCTGGTGTGTAATATCAACAAAGGCT
GTGGCTATGGCTGTCAGCTCCATCATGTGGTCTACTGCTTCATGATTGCATA
TGGCACCCAGCGAACACTCATCTTGGAATCTCAGAATTGGCGCTATGCTACT
GGTGGATGGGAGACTGTATTTAGGCCTGTAAGTGAGACATGCACAGACAGA
TCTGGCATCTCCACTGGACACTGGTCAGGTGAAGTGAAGGACAAAAATGTT
CAAGTGGTCGAGCTTCCCATTGTAGACAGTCTTCATCCCCGTCCTCCATATT
TACCCTTGGCTGTACCAGAAGACCTCGCAGATCGACTTGTACGAGTGCATG
GTGACCCTGCAGTGTGGTGGGTGTCTCAGTTTGTCAAATACTTGATCCGCCC
ACAGCCTTGGCTAGAAAAAGAAATAGAAGAAGCCACCAAGAAGCTTGGCTT
CAAACATCCAGTTATTGGAGTCCATGTCAGACGCACAGACAAAGTGGGAAC
AGAAGCTGCCTTCCATCCCATTGAAGAGTACATGGTGCATGTTGAAGAACA
TTTTCAGCTTCTTGCACGCAGAATGCAAGTGGACAAAAAAAGAGTGTATTT
GGCCACAGATGACCCTTCTTTATTAAAGGAGGCAAAAACAAAGTACCCCAA
TTATGAATTTATTAGTGATAACTCTATTTCCTGGTCAGCTGGACTGCACAAT
CGATACACAGAAAATTCACTTCGTGGAGTGATCCTGGATATACATTTTCTCT
CTCAGGCAGACTTCCTAGTGTGTACTTTTTCATCCCAGGTCTGTCGAGTTGCT
TATGAAATTATGCAAACACTACATCCTGATGCCTCTGCAAACTTCCATTCTT
TAGATGACATCTACTATTTTGGGGGCCAGAATGCCCACAATCAAATTGCCAT
TTATGCTCACCAACCCCGAACTGCAGATGAAATTCCCATGGAACCTGGAGA
TATCATTGGTGTGGCTGGAAATCATTGGGATGGCTATTCTAAAGGTGTCAAC
AGGAAATTGGGAAGGACGGGCCTATATCCCTCCTACAAAGTTCGAGAGAAG
ATAGAAACGGTCAAGTACCCCACATATCCTGAGGCTGAGAAATAACTCGAG
