# Supplementary material for: Structural basis for substrate specificity and catalysis of α1,6-fucosyltransferase
Source: Nat Commun. 2020 Feb 20;11:973. doi: 10.1038/s41467-020-14794-z (PMC7033129; doi:10.1038/s41467-020-14794-z)
Supplement: Supplementary file 2 — Peer Review File [file 41467_2020_14794_MOESM2_ESM.pdf]

Editorial Note: This manuscript has been previously reviewed at another journal that is not operating a transparent peer review scheme. The manuscript was considered suitable for publication without further review at *Nature Communications*.
